# Supplementary material for: Quantifying microbial fitness in high-throughput experiments
Source: eLife. 2026 Nov 2;13:RP102635. doi: 10.7554/eLife.102635 (PMC13727076; doi:10.7554/eLife.102635)
Supplement: Peer review history

# Editor report: eLife Assessment

**DOI:** 10.7554/eLife.102635.4.sa0

**Authors:** Sara Mitri

**Affiliations:** University of Lausanne Switzerland

**Keywords:** Convincing, Important

This manuscript applies a theoretical analysis to two published datasets on yeast and bacterial evolution to compare different ways of quantifying fitness. It makes an **important** advance by clarifying how discrepancies can arise by using different approaches and provides recommendations for best practices. Overall, this is an impressive and highly beneficial study that is based on **convincing** evidence and has the potential of setting standards in this rapidly growing field.

# Referee report: Reviewer #1 (Public review)

**DOI:** 10.7554/eLife.102635.4.sa1

[Editors' note: this version has been assessed by the Reviewing Editor without further input from the original reviewers. The authors have addressed the comments raised in the previous round of review.]

The authors point out that the fitness estimates obtained from different experimental assays (monoculture, pairwise competition or bulk competition) are not generally equivalent, not even with regard to the fitness ranking of different genotypes. Using a computational model based on experimentally measured growth phenotypes for knockout strains in yeast, as well as data from Lenski's Long Term Evolution Experiment (LTEE), they derive a set of best practice rules aimed at extracting the optimal amount of information from such experiments.

The study is very complete on a technical level, and the conceptual weaknesses raised in the first round of reviews have been fully addressed in the revision.

# Referee report: Reviewer #2 (Public review)

**DOI:** 10.7554/eLife.102635.4.sa2

Summary:

The manuscript "Quantifying microbial fitness in high-throughput experiments" provides a comprehensive analysis of the various approaches to quantifying fitness in microbial evolution, focusing on three primary factors: encoding of relative abundance, time scale of measurement, and the choice of reference subpopulation. The authors systematically explore how these choices impact fitness statistics and provide recommendations aimed at standardizing practices in the field. This manuscript aims to highlight the impact of differing fitness definitions and the methodologies utilized for analysis and how that can significantly alter interpretations of mutant fitness, affecting evolutionary predictions and the overall understanding of genetic interactions in the experiments.

Strengths:

The choices for quantifying fitness in evolution experiments are critical and highly relevant given the increasing prevalence of high-throughput experiments in evolutionary biology. The authors methodically categorize fitness statistics and their implications, providing clarity on a complex subject. This structured approach aids in understanding the nuances of fitness measurement. The manuscript effectively highlights how different choices in fitness measurement can influence fitness rankings and the understanding of epistasis, which is important for modeling evolutionary dynamics.

Comments on revisions:

The authors have comprehensively addressed all previous comments and suggestions. In particular, the addition of the new methods section: 'A guide to calculate pairwise relative fitness under the logit encoding from bulk competition data' - significantly improves the clarity of the implementation and helps in the overall interpretation of the framework.

# Referee report: Reviewer #3 (Public review)

**DOI:** 10.7554/eLife.102635.4.sa3

Summary:

The authors present analyses of different fitness measures derived from empirical data from yeast knock-out mutants and the long-term evolution experiment (LTEE) with *Escherichia coli* to explore discrepancies and identify preferred methods to estimate relative fitness in high-throughput experiments. Their work has three components. They first discuss the different "encodings" of relative abundance data and conclude that logit-transformations are preferred, because they transform nonlinear abundance trajectories into linear trajectories with greater predictive power. Next, they compare per-generation with per-growth cycle relative fitness estimates inferred from simulations of pairwise competitions based on published growth traits for the yeast strains and on published pairwise competition measurements for the LTEE data. Both data sets show quantitative and qualitative (i.e. rank order) discrepancies of estimates across different time scales, which are highlighted by considering possible underlying causes (i.e. trade-offs between growth traits) and consequences (i.e. epistasis among mutations affecting different growth traits). Finally, the authors compare simulated pairwise and bulk (i.e. where many mutants compete during a growth cycle in a single environment) competition assays based on the yeast knock-out mutants and demonstrate an optimal ratio of collective mutants to wild-type strains that minimizes both sampling error and overestimation of fitness estimates when compared with pairwise competitions.

Strengths:

The study deals with a highly relevant topic. Fitness is central to general evolutionary theory, but also poorly defined and implies different traits for different organisms and conditions. For microbes, which are often used in evolution experiments, high-throughput experiments may yield different measures to quantify abundance over time, from individual growth traits to bulk competition experiments. Hence, it is relevant to consider discrepancies among those measures and identify preferred measures with respect to predicting population dynamic and evolutionary processes. The present study contributes to this aim by (i) making readers aware of differences among commonly used fitness estimates, (ii) showing that simulated (yeast) and calculated (*E. coli*) competitive fitness may differ across time scales, and (iii) showing that bulk competitions may yield relative fitness estimates that are systematically higher than pairwise competitions. The study is rather thorough on the theory side, with extensive derivations and analyses of various fitness measures using their resource competition model in the Supplementary Information. The study ends with a few practical recommendations for preferred methods to infer relative fitness estimates, that may be useful for experimentalists and stimulate further investigations.

Comments on revisions:

I appreciate the thorough and effective response to all recommendations and have no further comments.

# Author comment: Author response

**DOI:** 10.7554/eLife.102635.4.sa4

**Authors:** Justus Wilhelm Fink, Michael Manhart

**Affiliations:** California Institute of Technology Pasadena United States; Rutgers, The State University of New Jersey Piscataway United States

The following is the authors’ response to the previous reviews

We thank both editors and the three reviewers for their positive feedback on the revised manuscript. Following suggestions from Reviewer #1, we cite additional literature to clarify the use of ’fitness potential’ and we have revised the caption of Figure 3 to better explain how we generate the panel of mutants. We have also added a sentence to emphasize that the selection coefficient should match the time-scale of bottleneck effects in the evolution environment.
